# Botanical Description of the Benjamin Tree of Sumatra

**Published:** 1788

**Authors:** Jonas Dryander

**Affiliations:** Member of the Royal Academy of Sciences at Stockholm


XI. *Botanical Defcription of the Benjamin Tree of Sumatra. By* Jonas Dryander, *M. A. Libr. R. S. and Member of the Royal Academy of Sciences at Stockholm; communicated by Sir* Jofeph Banks, *Bart. P. R. S.*—From the *Philofophical Tranfactions of the Royal Society of London,* Vol. LXXVII, *for the Year* 1787. Part II. 4to. London, 1787.

THOUGH Garcias ab Horto, Grim, and Sylvius, were acquainted with the real tree from which Benjamin or Benzoin is collected, their defcriptions of it are fo imperfect and infufficient for its botanical determination, that fucceeding botanifts have fallen into into many errors concerning it; and it is remarkable, that although this drug was always imported from the Eaſt-Indies, moſt of the later writers on the Materia Medica have conceived it to be collected from a ſpecies of Laurus, native of Virginia, to which, from this erroneous ſuppoſition, they have given the trivial name of Benzoin. This miſtake ſeems to have originated with Mr. Ray, who in his Hiſtoria Plantarum, Vol. II. p. 1845, at the end of his account of the Arbor Benivifera of Garcias, ſays: "Ad nos ſcripſit D. Tancredus "Robinſon Arborem reſiniferam odoratam foliis citrinis prædictæ haud abſimilem tranſmiſſam fuiſſe e Virginia a D. Baniſter, ad "illuſtriſſimum Præſulem D. Henr. Compton, "in cujus inſtructiſſimo horto culta eſt.—— "Arbor iſta Virginiana Citrii, vel Limonii foliis Benzoinum fundens, in horto reverendiſſimi Epiſcopi culta."

This error was detected by Linnæus, but another was ſubſtituted by him in its place; for in his Mantiſſa Plantarum Altera, he tells us, that Benjamin is furniſhed by a ſhrub deſcribed there under the name of Croton Benzoe, and afterwards in the Supplementum Plantarum, deſcribes again the ſame plant, under the name of Terminalia Benzoin. Mr. Jacquin, who had been informed that this shrub was called by the French *Bienjoint*, supposes, with reason, that the similar sound of that word with *Benjoin*, the French name for Benjamin, may have occasioned this mistake*.

Since that period Dr. Houttuyn has described the Benjamin Tree of Sumatra; but for want of good specimens, has been so unfortunate as to mistake the genus to which it belongs. It is hoped, therefore, that the following description and annexed figure †, may not be unworthy a place in the Philosophical Transactions; they are made from dried specimens procured from Sumatra by Mr. Marsden, F.R.S. at the request of Sir Joseph Banks, Bart. P.R.S. and clearly prove that this tree agrees in the parts of fructification with the Styrax of Linnæus.

STYRAX *Benzoin*, foliis oblongis acuminatis subtus tomentosis, racemis compositis longitudine foliorum.

Benjui. *Garcias ab Horto* in *Clusii Exoticis, p.* 155.

* Hort. Vindob. Vol. III. p. 51.

† For the figure we must refer our readers to the Philosophical Transactions.—EDITOR.

Arbor

Arbor Benzoini. *Grim* in *Ephemer. Acad. Nat. Curiof. Dec.* 2. *Ann. I. p.* 370. *fig.* 31. *Sylvius* in *Valentini Hiftoria Simplicium, p.* 487.

Benzuin. *Radermacher* in *Act. Societ. Bataviæ, vol. III. p.* 44.

Benjamin or Benzoin. *Marfden's Hift. of Sumatra, p.* 123.

Laurus Benzoin. *Houttuyn* in *Act. Harlem. vol. XXI. p.* 265. *tab.* 7.

*Habitat* in Sumatra. ♄

### DESCRIPTIO.

*Rami* teretes, tomentofi.

*Folia* alterna, petiolata, oblonga, integerrima, acuminata, venofa, fupra glabra, fubtus tomentofa, palmaria. *Petioli* teretes, ftriati, canaliculati, tomentofi, breviffimi.

*Racemi* axillares, compofiti, longitudine fere foliorum. *Pedunculi communes* tomentofi; *partiales* alterni, patentes, tomentofi. *Pedicelli* breviffimi. *Flores* fecundi.

*Calyx* campanulatus, obfoletiffime quinquedentatus, extus tomentofus, linea longior.

*Petala* quinque, (bafi forte connata) linearia, obtufa, extus tomento tenuiffimo cinerea, calyce quadruplo longiora.

*Filamenta* decem, receptaculo inserta, petalis paulo breviora, inferne connata in cylindrum longitudine calycis, superne infra antheras ciliata. *Antheræ* lineares, filamentis longitudinaliter adnatæ, iisque dimidio breviores. *Germen* superum, ovatum, tomentosum. *Stylus* filiformis, staminibus longior. *Stigma* simplex.

